# Supplementary material for: An image analysis pipeline to quantify the spatial distribution of cell markers in stroma-rich tumors
Source: bioRxiv. 2025 May 1:2025.04.28.650414. Preprint. [Version 1] doi: 10.1101/2025.04.28.650414 (PMC12247833; doi:10.1101/2025.04.28.650414)
Supplement: Supplement 1 [file media-1.pdf]

Supplementary for:

An image analysis pipeline to quantify the spatial distribution of cell markers in stroma-rich tumors

Antoine A. Ruzette[1], Nina Kozlova[2,3], Kayla A Cruz[2,4], Taru Muranen[2,3], and Simon F. Nørrelykke[1*]

Affiliations:

1) Department of Systems Biology, Harvard Medical School, Boston, Massachusetts, USA
2) Department of Genetics, Cancer Research Institute, Beth Israel Deaconess Medical Center, Boston, Massachusetts, USA
3) Harvard Medical School, Boston, Massachusetts, USA
4) Biological and Biomedical Sciences PhD Program, Harvard University, Boston, Massachusetts, USA

* Correspondence: simon@hms.harvard.edu

Paper website: https://hms-iac.github.io/stroma-spatial-analysis-web

Supplementary tables and figures

Numbers in tables were rounded up to the sixth significant decimal.

AsPC NDRG1

Table S1: Parameters of the fitted lognormal distribution for the ***FITC KER: Cytoplasm: Median*** intensity across all detected cells. The intensity threshold used for cell classification in each image is determined by translating the reference threshold percentile (marked with an *).

Image	Location	Shape	Scale	Threshold
**#1**	–707.011083	0.619916	3138.058254	300*
**#2**	–723.643241	0.603754	2949.356883	251.28
**#3**	–793.205413	0.618143	3229.167415	246.42
**#4**	–308.258962	0.681428	1840.263228	219.30
**#5**	–1129.335995	0.558484	4068.679791	331.98

Table S2: Parameters of the fitted lognormal distribution for the ***CY5 pNDRG1: Cell: Max*** intensity across all detected cells. The intensity threshold used for cell classification in each image is determined by translating the reference threshold percentile (marked with an *)

Image	Location	Shape	Scale	Threshold
**#1**	734.582782	1.361657	1067.563348	1200*
**#2**	700.593146	1.238082	756.449602	1056.18
**#3**	703.018174	1.229991	828.940911	1094.61
**#4**	687.089880	1.151794	608.544201	988.61
**#5**	721.599776	1.516394	1077.707109	1149.14

Table S3: Parameters of the fitted lognormal distribution for the ***TRITC FN: Cell: Median*** intensity across all detected cells. The intensity threshold used for cell classification in each image is determined by translating the reference threshold percentile (marked with an *)

Image	Location	Shape	Scale	Threshold
**#1**	1127.574751	0.680330	1557.668026	5000*
**#2**	1073.706558	0.741478	1482.000040	5072.28
**#3**	913.571101	0.573779	1331.199930	3783.08
**#4**	884.531585	0.670759	876.069113	3034.75
**#5**	1276.548976	0.727041	1644.706573	5629.18

AsPC Ki67

Table S4: Parameters of the fitted lognormal distribution for the ***FITC KER: Cytoplasm: Median*** intensity across all detected cells. The intensity threshold used for cell classification in each image is determined by translating the reference threshold percentile (marked with an *)

Image	Location	Shape	Scale	Threshold
**#1**	288.717525	0.599332	1632.425859	650*
**#2**	-550.184474	0.564812	4726.485901	590.79
**#3**	-9.668936	0.489416	2167.226010	622.80
**#4**	-310.003757	0.416821	2126.460683	434.95
**#5**	-340.941994	0.797041	8712.196935	831.45

Table S5: Parameters of the fitted lognormal distribution for the ***CY5 Ki67: Nucleus: Max*** intensity across all detected cells. The intensity threshold used for cell classification in each image is determined by translating the reference threshold percentile (marked with an *).

Image	Location	Shape	Scale	Threshold
**#1**	286.724866	1.801891	469.496465	950*
**#2**	282.490001	1.643360	474.312730	932.51
**#3**	348.348119	1.593198	752.831402	1370.18
**#4**	241.491744	1.597186	255.996654	589.23
**#5**	260.713033	1.705415	707.838623	1242.37

Table S6: Parameters of the fitted lognormal distribution for the ***FITC FN: Cell: Median*** intensity across all detected cells. The intensity threshold used for cell classification in each image is determined by translating the reference threshold percentile (marked with an *).

Image	Location	Shape	Scale	Threshold
**#1**	695.439669	0.477924	1499.172311	4000*
**#2**	1053.805650	0.663097	1866.380304	6641.85
**#3**	1258.215495	0.603436	1219.408835	4566.16
**#4**	564.392175	0.634543	718.958016	2617.70
**#5**	805.830558	0.649840	1221.342242	4383.30

Table S7: Agreement percentages of single-threshold classifiers when compared to machine learning classification results for pNDRG1 images. The same thresholds from one image were applied to classify cells in other images. σ is the sample standard deviation. All values are in percentage (%).

**ML classifier**	**Classifier using thresholds from image number:**				
	**#1**	**#2**	**#3**	**#4**	**#5**
87.7	96.1	86.4	90.1	78.7	94.0
	μ = 89.06<br>σ = 6.88				

Table S8: Agreement percentages of single-threshold classifiers when compared to machine learning classification results for Ki67 images. The same thresholds from one image were applied to classify cells in other images. σ is the sample standard deviation. All values are in percentage (%).

**ML classifier**	**Classifier using thresholds from image number:**				
	**#1**	**#2**	**#3**	**#4**	**#5**
86.3	84.2	83.7	92.3	66.6	90.4
	μ = 83.4<br>σ = 10.1				

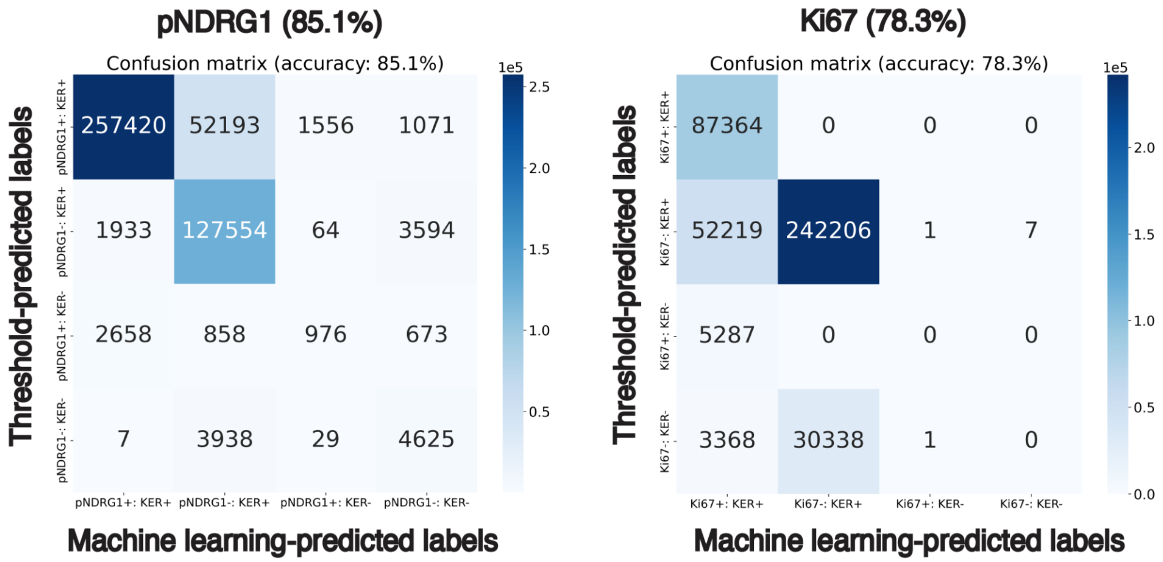

Figure S1: Comparison of machine learning-based and threshold-based classifiers across all possible class assignments from the following four classes: KER+, KER-, pNDRG1- or Ki67+, and pNDRG1- or Ki67-.

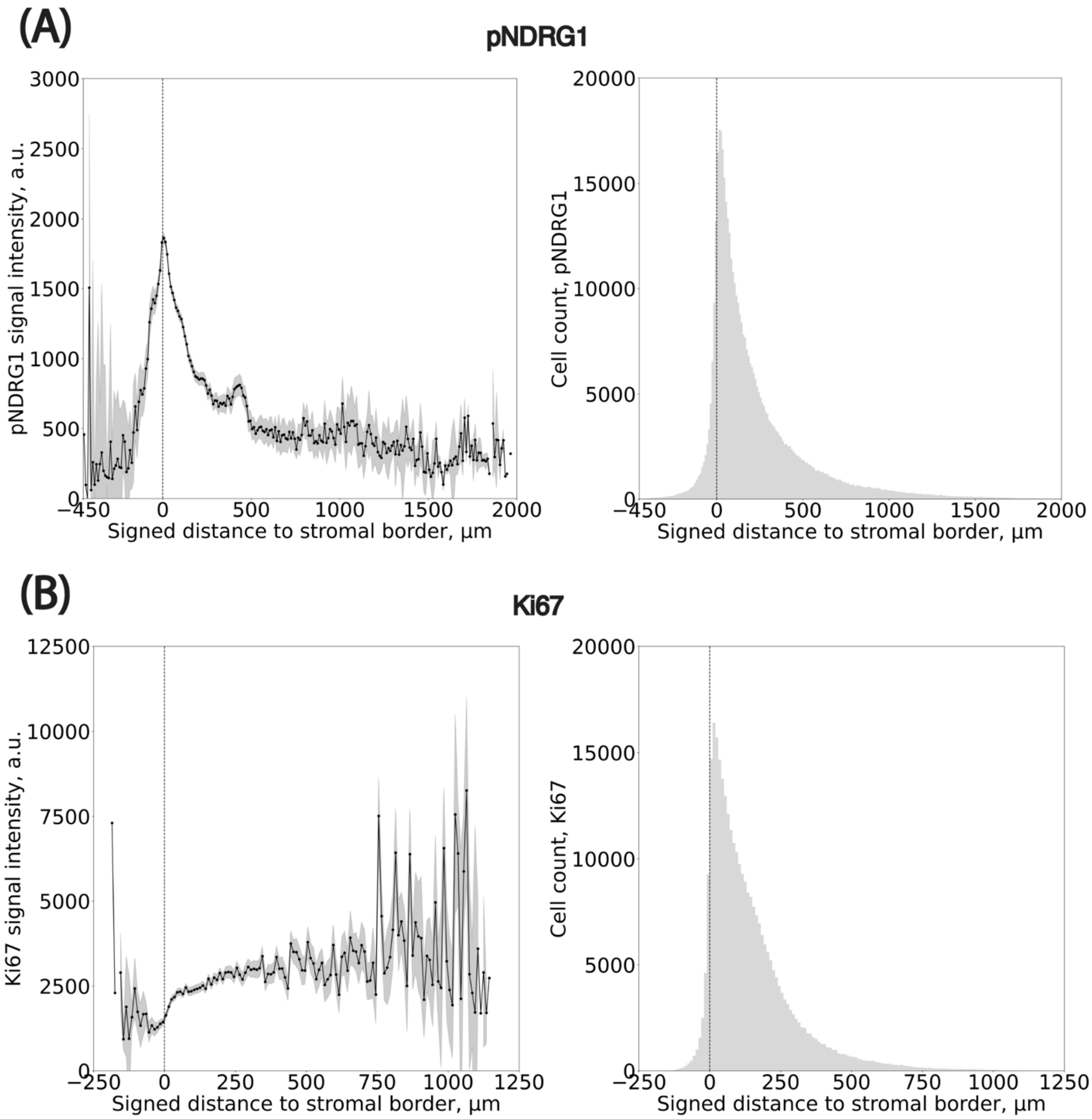

Figure S2: Full spatial distribution of double positive cells (cytokeratin positivity and pNDRG1 or Ki67) relative to the modelled stromal border.
(A) (left) Full spatial distribution of pNDRG1-positive cancer cells. Bin size: 10 μm. x axis is unclipped. Black dots represent the median value within a bin. Grey overlays indicate Standard Error of the Mean [SEM]. (right) Number of cells per bin. Same bin size. **(B)** Same plots repeated for Ki67-positive cancer cells. Bin size: 10 μm.
